# Hypothesis: The Intratumoral Immune Response against a Cancer Progenitor Cell Impacts the Development of Well-Differentiated versus Dedifferentiated Disease in Liposarcoma

**DOI:** 10.3389/fonc.2016.00134

**Published:** 2016-06-10

**Authors:** William W. Tseng, Shefali Chopra, Edgar G. Engleman, Raphael E. Pollock

**Affiliations:** ^1^Section of Surgical Oncology, Department of Surgery, Keck School of Medicine, University of Southern California, Los Angeles, CA, USA; ^2^Sarcoma Program, Hoag Family Cancer Institute, Hoag Memorial Hospital Presbyterian, Newport Beach, CA, USA; ^3^Department of Pathology, Keck School of Medicine, University of Southern California, Los Angeles, CA, USA; ^4^Department of Pathology, Stanford University School of Medicine, Palo Alto, CA, USA; ^5^Division of Surgical Oncology, Department of Surgery, The James Comprehensive Cancer Center, Ohio State University, Columbus, OH, USA

**Keywords:** liposarcoma, dedifferentiation, tertiary lymphoid structures, ectopic lymph node, cancer stem cells, tumor-initiating cells

## Abstract

Well-differentiated/dedifferentiated (WD/DD) liposarcoma is a rare malignancy of adipocyte origin (“fat cancer”). Tumors may be entirely WD, WD with a DD component, or rarely DD without a clear WD component. WD tumors are low grade and generally indolent, while tumors with a DD component are high grade and behave much more aggressively, with a modest potential for distant metastasis. The presence of cancer progenitor cells in WD/DD liposarcoma is suggested by clinical evidence and reported research findings. In addition, there are emerging data to support the existence of a naturally occurring, antigen-driven, and adaptive immune response within the tumor microenvironment. We hypothesize that the intratumoral immune response is directed against a cancer progenitor cell and that the outcome of this response impacts the development of WD versus DD disease. Further study will likely provide interesting insights into the disease biology of WD/DD liposarcoma that may be readily translated to other more common cancers.

Well-differentiated/dedifferentiated (WD/DD) liposarcoma is a rare form of cancer, which originates from an adipocyte and is therefore a malignancy of fat (1, 2). Tumors most commonly occur in the retroperitoneum, where they can reach impressive size (mean: 30 cm) by the time of initial presentation (3, 4). In addition, unique to this disease, tumors can have distinct low-grade (WD) and high-grade (DD) areas juxtaposed next to one another. In fact, tumors may be entirely WD, WD with a DD component, or rarely DD without a clear WD component. The true relationship between the WD and DD components of tumor is unclear; however, presence of DD dramatically changes the disease biology and clinical outcome (1, 2, 5).

In this article, we will review available data to support the presence of cancer progenitor cells and an intratumoral immune response in this disease. Merging these two areas of ongoing research, we propose a unique hypothesis that may help to elucidate disease biology in WD/DD liposarcoma. We believe that the immune response is directed against a cancer progenitor cell and that the outcome of this response impacts the development of WD versus DD disease.

## WD and DD: “A Good Brother and a Bad Brother”

Both WD and DD liposarcoma share a common genetic aberration, amplification of chromosomal region 12q13–15 (1, 2). This results in up to several thousand copies of a number of critical genes, including MDM2 and CDK4. Amplification of this region and/or specific genes found in this region (e.g., MDM2) can be detected by fluorescence *in situ* hybridization (FISH), and this test is used to establish diagnosis of WD/DD liposarcoma in cases in which diagnosis cannot be made on histology alone.

**Figure 1:**
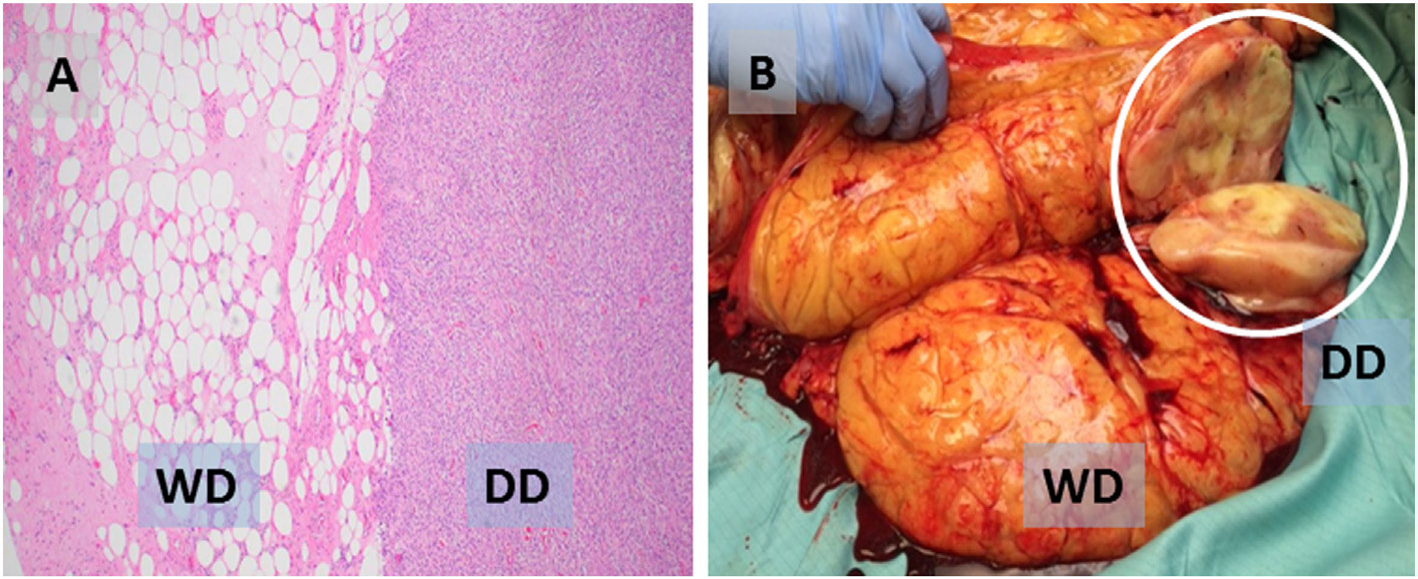
**(A)** Photomicrograph (H&E) showing characteristic liposarcoma histology with juxtaposed well-differentiated (WD, low grade) and dedifferentiated (DD, high grade) areas of the same tumor. **(B)** Sectioned WD tumor with a probable focus of DD (white circle).

Despite common genetic features, WD and DD liposarcoma can be thought of as a spectrum of disease with clear differences between WD and DD (5). By histology, WD liposarcoma (low grade) consists of adipocytes of varying size, separated by fibrous bands with hyperchromatic cells (HCs); in contrast, in DD liposarcoma (high grade), the adipocytic areas are replaced by cellular areas, frequently with mitotic figures (Figure 1A). Macroscopically, WD tumors contain mostly fat, whereas DD areas of tumor are more dense, with a white, “fleshy,” and/or vascularized appearance (Figure 1B). These differences can be seen on cross-sectional imaging, which can suggest the presence of DD (often seen arising from or adjacent to WD areas of tumor); however, the final diagnosis of DD is made by histology.

Tumors that have a DD component are clearly more aggressive, and patients overall do worse. WD tumors are generally indolent and do not have the potential for distant metastasis. In contrast, tumors with DD frequently invade into adjacent organs and structures and up to 20–30% of cases metastasize, most commonly to the lungs (6). After surgery, the rates of locoregional recurrence are higher, and the time of recurrence is faster in DD versus WD tumors (7, 8). Interestingly, although response to radiation therapy and systemic therapy is overall poor for both, for cytotoxic chemotherapy (e.g., doxorubicin), DD has a slightly better response rate compared to WD (12 versus 0%) (9).

## Cancer Progenitor Cells in WD/DD Liposarcoma

Cancer progenitor cells or “stem cells” have been reported and characterized in a number of human solid tumors (10–13). This unique subpopulation of cells is defined by the ability to self-renew and the capacity to differentiate into other cell types (pluripotency). Cancer progenitor cells are tumorigenic and, even in small numbers, can form an entire tumor. Clinically, these cells are thought to be more resistant to therapy compared to non-progenitor cells. As a result, cancer progenitor cells may play a fundamental role not only in tumorigenesis but also in recurrence and even distant metastasis. Specific cell surface and functional markers have been identified, which are suggestive of cancer progenitor cells.

There is some clinical evidence to suggest the presence of a cancer progenitor cell in WD/DD liposarcoma. Evans et al. was one of the first to report the observation of heterologous differentiation in this disease (14). By histology, some tumors (~5–10%) may exhibit features consistent with lineage differentiation to bone, muscle, blood vessel, or other tissue, suggesting the existence of a cell within the tumor with pluripotency (15, 16). WD/DD liposarcoma has also been reported to express CD117 or c-kit, also known as “stem cell factor” (17, 18). In addition, some tumors have high expression of CD34, another established stem cell marker (19). Riddle et al. even described a case report of a patient with a CD117 and CD34 double positive, but MDM2-amplified, liposarcoma “masquerading” as a gastrointestinal stromal tumor (20).

Several research investigators have also directly explored the presence of cancer progenitor cells in WD/DD liposarcoma. Stratford et al. used immunohistochemistry (IHC) to show that all (six out of six) cases of WD/DD liposarcoma expressed high levels of aldefluor, a functional stem cell marker (21). Interestingly, all the cases were also negative for CD133, a different stem cell marker. Using a human liposarcoma cell line (SW872)-based xenograft mouse model, the authors were able to identify a progenitor cell subpopulation that could form *de novo* tumors with as few as 100 cells. Smith et al. studied a xenograft mouse model established by implanting human WD/DD liposarcoma tumors obtained from surgery. Tumors that engrafted successfully appeared to have a gene signature with a “progenitor-like phenotype” (22).

It is important to note that the presence of cancer progenitor cells seems to be more pronounced in DD than WD liposarcoma. Heterologous differentiation is mostly seen in DD. CD117 expression was seen in 30% of DD cases studied by Tayal et al. versus none in WD (17). In liposarcoma cell lines, the majority of those with evidence for stem cell potential are DD or derived from “poorly differentiated” tumors (23). In xenograft mouse models, both Smith et al. and Peng et al. have independently confirmed that DD but not WD tumors have the potential to engraft successfully (22, 24). As a likely explanation, the highest percentage of CD34-positive stem cells is seen in DD tumors compared to WD and normal fat (25).

## The Intratumoral Immune Response in WD/DD Liposarcoma

**Figure 2:**
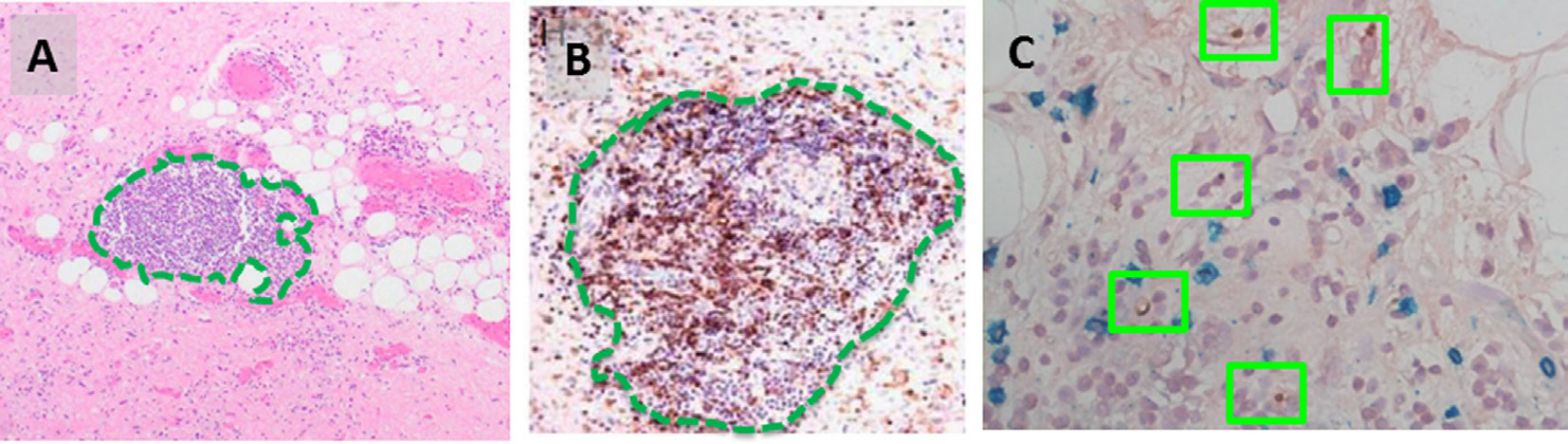
**(A)** Intratumoral tertiary lymphoid structure (TLS, green outline) found in WD liposarcoma. **(B)** By immunohistochemistry, TLS contain a dense population of CD4 T cells (brown). **(C)** DC-LAMP-positive dendritic cells are found adjacent to CD4 T cells (green boxes).

In the late 1990s, Argani et al. and Kraus et al. originally described an “inflammatory” variant of WD liposarcoma, based on the presence of a prominent immune infiltrate (26, 27). Recently, our group has reported a more detailed characterization of the intratumoral immune response that occurs naturally in both WD and DD liposarcoma (28, 29). One noteworthy observation is the presence of organized aggregates of immune cells within the tumor microenvironment, known as tertiary lymphoid structures (TLS) (Figure 2). Our work demonstrated that TLS contain mature dendritic cells situated adjacent to CD4 helper T cells, a feature suggestive of antigen presentation. TLS also contain B cells and, in fact, can have a clear germinal center. CD8 cytotoxic T cells are also found; however, these are typically scattered in the areas of tumor outside of TLS. Taken together, our findings strongly suggest that there is an antigen-driven, adaptive immune response in WD/DD liposarcoma. The key question is: what are the tumor antigen(s) that are being targeted by the intratumoral immune response in WD/DD liposarcoma?

## Stromal Hyperchromatic Cells: The “Missing Link”?

Within the tumor stroma of WD liposarcoma, there exists an atypical, spindle-shaped cell with a hyperchromatic nucleus. These HCs are seen often enough by routine histology that they are used by clinical pathologists as part of the criteria for disease diagnosis (30, 31). In support of this, by FISH, HCs demonstrate high, if not the highest levels of amplification, confirming their identity as tumor cells (32). However, the significance of HCs in WD/DD liposarcoma has never been studied to our knowledge.

**Figure 3:**
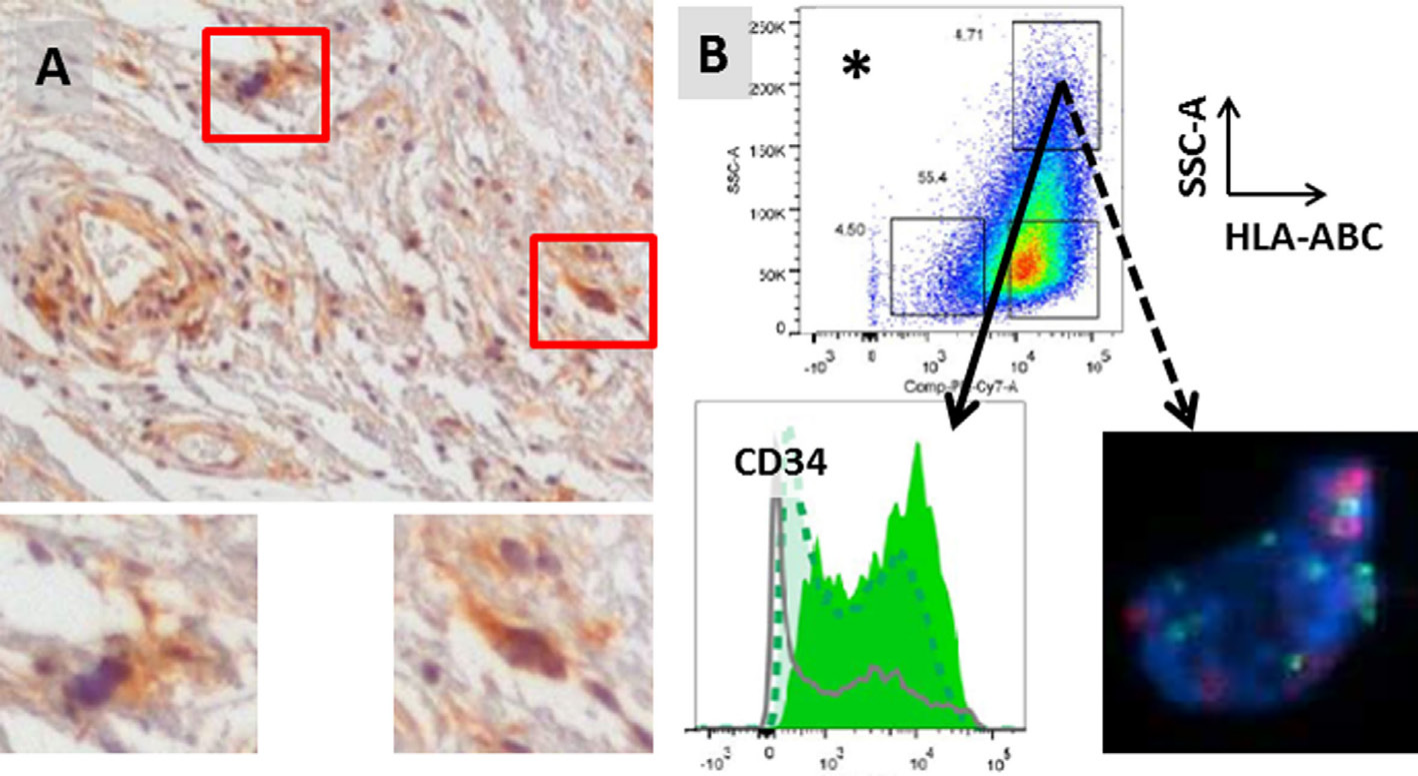
**(A)** Stromal hyperchromatic cells (HCs) in WD/DD liposarcoma (red boxes). HCs express MHC Class I (brown, insets). **(B)** HCs also express high levels of CD34 (solid arrow) and have 12q13–15 amplification (dotted arrow; FISH courtesy of KL Bill). *HCs were isolated from fresh tumor tissue after excluding immune cells and endothelial cells and gating on the population with the highest side scatter (SSC-A = internal complexity) and Class I (HLA-ABC).

Our group has preliminary data to suggest that HCs express both CD34 and MHC Class I (Figure 3). CD34 has been reported to be a marker for adipocyte stem cells (ASCs) by several investigators. In addition to liposarcoma (19, 20, 25), CD34-positive cells with stem cell gene expression and functional characteristics have also been reported in normal fat and benign lipomas (33–36). Although further studies are needed, our preliminary data would suggest that HCs may be ASCs in WD/DD liposarcoma. MHC Class I is a cell surface molecule involved in antigen display. Although tumor cells can downregulate MHC Class I to evade immune detection, its expression on HCs suggests that HC antigen can be recognized by the adaptive immune response.

## Hypothesis and Further Areas of Study

**Figure 4:**
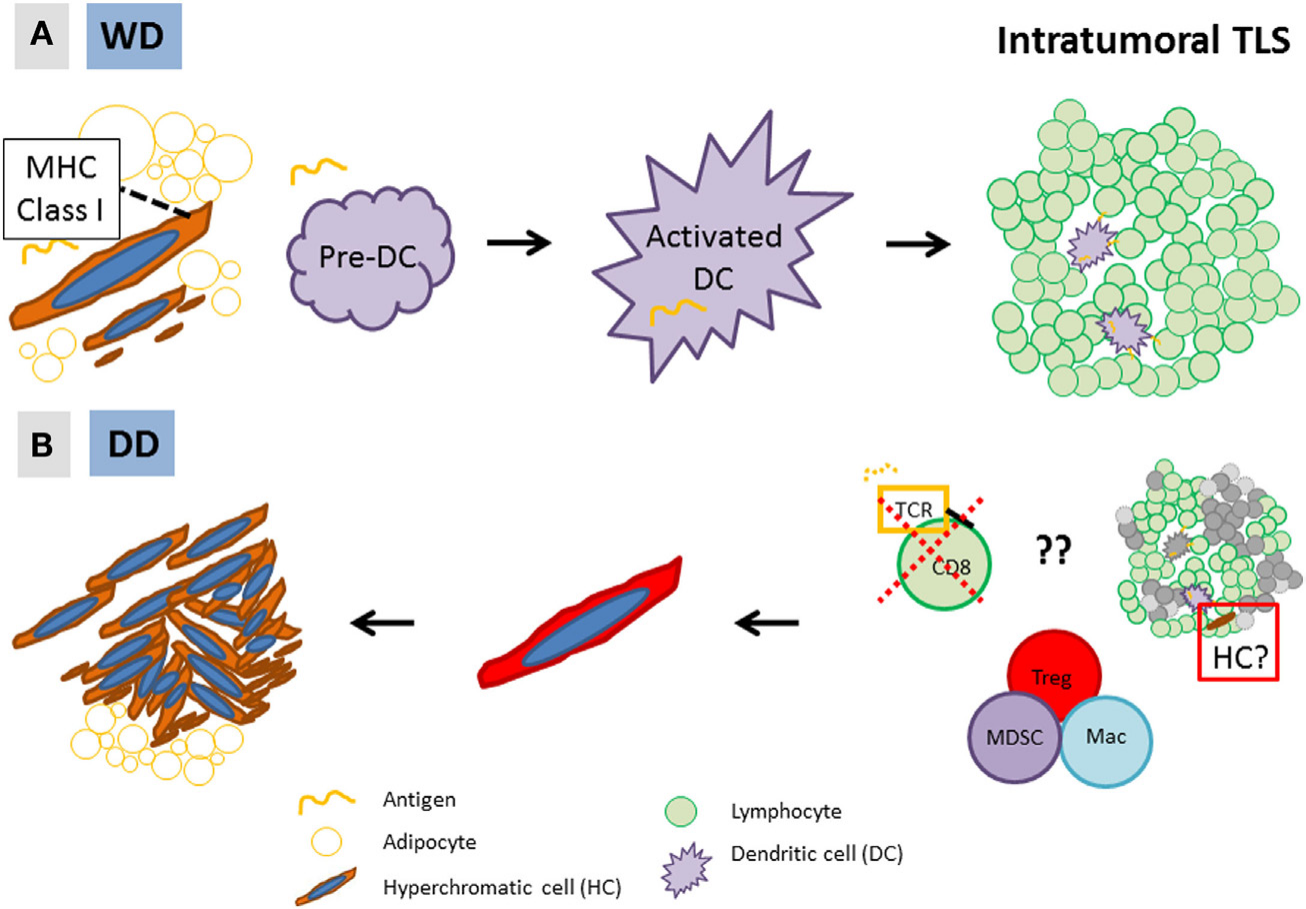
Hypothesis: **(A)** in low-grade, well-differentiated (WD) disease, MHC Class I-expressing stromal hyperchromatic cells (HCs) differentiate along an adipocytic lineage and undergo slow proliferation. Some HCs are recognized by dendritic cells (DC), which form an intratumoral tertiary lymphoid structure (TLS); **(B)** however, through yet undefined mechanisms, HCs are able to evade the immune response and rapidly proliferate, resulting in high-grade, dedifferentiated (DD) disease. Alternatively, TLS may be an “immune-privileged” site that directly supports the growth of HCs (right, red box).

We hypothesize that, in WD/DD liposarcoma, the intratumoral immune response (TLS) is directed against a candidate cancer progenitor cell (HC). We further hypothesize that differential immune responses against this unique tumor cell ultimately leads to the development of WD versus DD disease (Figure 4). Initially, HCs undergo a low level of proliferation that results in a WD tumor. On a cellular level, HCs (CD34-positive, likely ASC) differentiate along an adipocytic lineage, and the histology is low grade. In at least some HCs, antigen is presented on the cell surface (*via* MHC Class I) and recognized by the adaptive immune cells, which, in response, form an intratumoral TLS. An attempt is made by the immune cells to eliminate HCs; however, this is unsuccessful. HCs are able to evade the adaptive immune response or alternatively, TLS may even become an “immune-privileged” site in direct support of HCs. The net result is much more rapid proliferation of HCs, forming the cellular areas characteristic of high-grade disease (=DD). The tumor microenvironment(s) in WD/DD liposarcoma may therefore represent a dynamic form of cancer immunoediting (“elimination, equilibrium, and escape”), similar to that which has been proposed by others (37–39). In WD/DD liposarcoma, immunoediting occurs across the spectrum of low- to high-grade disease and is directed against a cancer progenitor cell.

**Figure 5:**
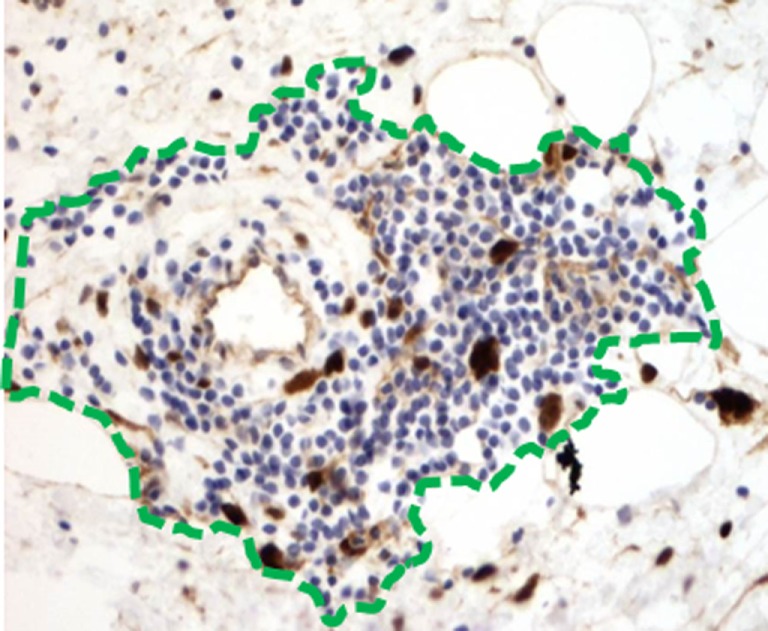
**Hyperchromatic and MDM2-positive tumor cells (brown) are found within and just adjacent to TLS (green outline)**.

**Figure 6:**
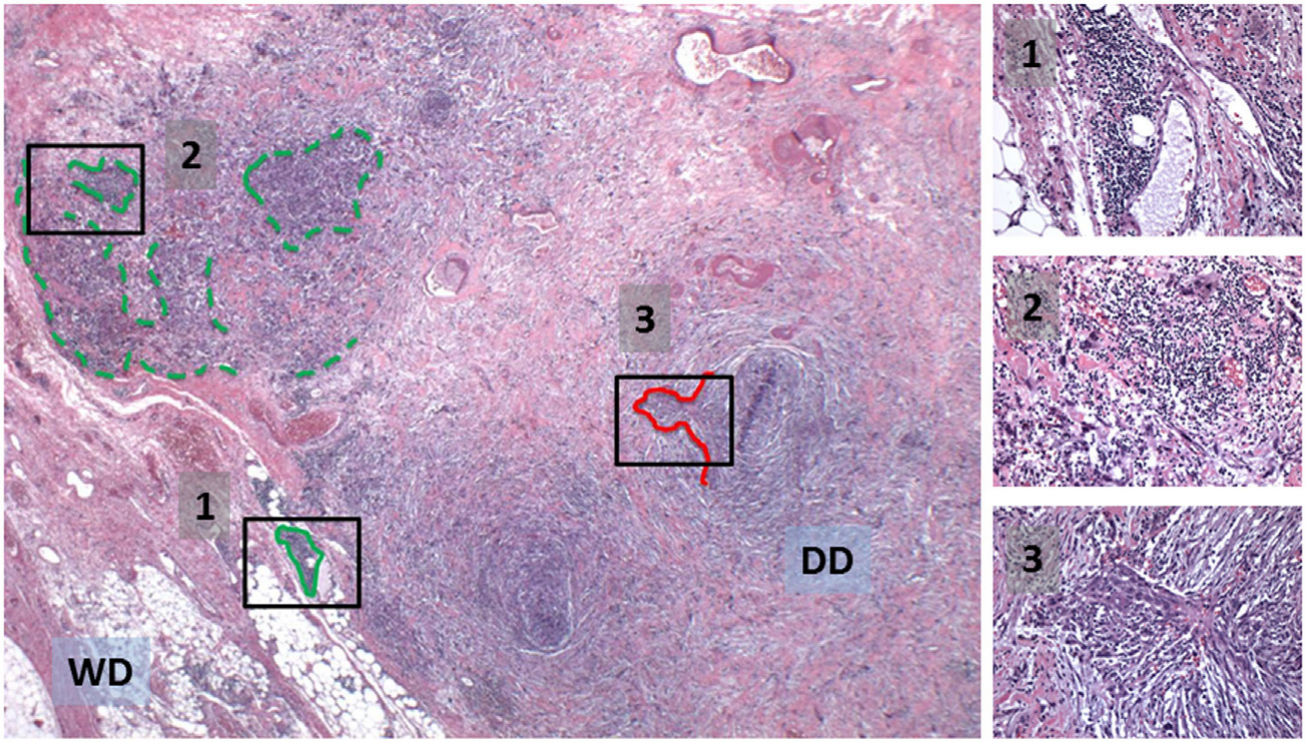
**Potential natural progression from a formed TLS (1, green outline) to disrupted TLS with an adjacent, developing cellular/DD area of tumor (2) to a locally-invasive, cellular/DD area (red outline) without TLS (3)**. See also insets.

Tertiary lymphoid structures have recently been suggested to serve as a microniche to promote the growth of cancer progenitor cells in hepatocellular carcinoma (HCC) (40). In the precancerous liver of a genetically engineered mouse model of HCC, TLS form around a progenitor cell, which proliferates and ultimately “overtakes” the TLS, resulting in frank HCC. The concept of progenitor cells existing in an immune-privileged niche has been reported in other cancers as well (41, 42). Re-examination of WD/DD liposarcoma demonstrates that, in fact, hyperchromatic and strongly MDM2-positive cells are found within TLS (Figure 5). Moreover, we have observed unique patterns of TLS organization that could be interpreted as a progressive “overtaking” of TLS by a developing cellular/DD area of tumor (Figure 6). Coincidentally, although TLS have been reported in a number of other cancers and shown to be associated with better prognosis, the opposite is true in both HCC and WD/DD liposarcoma (29, 40).

Further study to support or refute our hypothesis is clearly needed. From an immunology standpoint, this should first occur broadly, looking at the differences in the immune response across the spectrum of disease, with tumors that are entirely WD, WD with a DD component, and DD without a WD component. If our hypothesis is true, the overall immune bias (cellular composition, function, and cytokine production) should differ vastly from WD to DD, even regionally within the same tumor. From a tumor standpoint, HCs should be isolated and further characterized. If HCs are the targets of TLS *via* antigen display on MHC Class I, it is unclear why they are selectively targeted. CD34-positive ASCs have been reported to undergo proliferation in benign lipomas (35, 36). In WD liposarcoma, it is possible that in some proliferating HCs low levels of mutated antigen(s) arise, which are then recognized by the adaptive immune cells. There should also be focused investigation into the initial cell-to-cell interaction between HCs and the dendritic cell precursors that will result in antigen presentation to naive T and B cells within TLS. Importantly, is there an ultimate failure of HC-antigen presentation or is the deficiency at the downstream effector cells targeting HCs? In support of the latter, we have previously reported that in WD/DD liposarcoma, intratumoral CD8 T cells, which are typically outside of TLS, have high expression of PD-1, an immune checkpoint molecule and natural “stop signal” for these effector cells (29). Alternatively, do the HCs recruit suppressive immune cell types or do they themselves directly suppress the mounting immune response? In support of the former, we recently described a case report of multicystic DD liposarcoma with a prominent monocytic immune cell subpopulation that is likely comprised of myeloid-derived suppressor cells and tumor-associated macrophages (43).

In WD/DD liposarcoma, further study of the intratumoral immune response against a candidate cancer progenitor cell will likely provide meaningful insights into the biology of this disease. However, one important caveat is that the clonal origin and true relationship between WD and DD is still unknown. Nonetheless, this work may also result in identification of clinical biomarkers for disease progression and novel targets for therapy (e.g., immunotherapy) (44). There may be a specific gene or cytokine signature in TLS that is indicative of pure WD versus early (subclinical) development of DD disease. One interesting observation is that DD occurs much less frequently in tumors that arise in the extremity and trunk compared to the retroperitoneum (15, 45, 46). Could this be driven or, at least, influenced by the intratumoral immune response (e.g., frequency or type of TLS formed)? In terms of therapy, appropriate manipulation of TLS may potentially prevent DD and maintain WD, which would improve prognosis and is overall a more clinically manageable disease.

Finally, further hypothesis-driven study in WD/DD liposarcoma may uncover findings that can be readily translated into other, more common cancers. There is a plethora of literature about cancer progenitor cells in a variety of cancers; there is now also an increasing body of work characterizing TLS in non-small cell lung cancer, melanoma, colorectal cancer, and others (47). To our knowledge, the true “targets” of the adaptive immune response represented by TLS in these cancers is still yet undefined. Therefore, although rare, with its unique disease characteristics, WD/DD liposarcoma may be an ideal “cancer model” to study the tumor–immune microenvironment and, specifically, from the standpoint of the immune response against cancer progenitor cells.

## Author Contributions

WWT and SC conceived of the hypothesis and reviewed the literature. EGE and REP provided critical discussion and input to help develop the hypothesis. WWT wrote the manuscript.

## Conflict of Interest Statement

The authors declare that the research was conducted in the absence of any commercial or financial relationships that could be construed as a potential conflict of interest.
